# Is Red the New Black? A Quasi-Experimental Study Comparing Perceptions of Differently Coloured Cycle Lanes

**DOI:** 10.3389/fpsyg.2020.554488

**Published:** 2020-12-04

**Authors:** Katrine Karlsen, Aslak Fyhri

**Affiliations:** Department of Security, Safety and Behaviour, Institute of Transport Economics, Oslo, Norway

**Keywords:** coloured cycle lane, web survey, perceived safety, inviting to cycle, visibility

## Abstract

Cities and road authorities in many countries have started colouring their cycle lanes. Some road authorities choose red, some blue, and some green. The reasoning behind this choice is not clear, and it is uncertain whether some colours are superior to others. The current study aims to examine whether coloured cycle lanes are viewed more positively than uncoloured lanes, and whether one of the typically chosen colours is perceived as safer and more inviting to cyclists or more deterring to motorists. Participants were invited to respond to a web survey. Respondents (*N* = 560) were from the four largest cities in Norway, both genders (42.1% women), and of a wide age range (79.6% between 30 and 70). Depending on cycling frequency, respondents were categorised as either motorist (*n* = 354) or cyclist (*n* = 206). All respondents rated different cycle lanes (uncoloured, blue, green, and red) on different attributes. The uncoloured lane was consistently rated least positively, with the lowest scores on visibility, perceived safety for both motorists and cyclist and how inviting it seemed to cycle in the lane. It was also estimated to be the lane that would experience the greatest degree of violation from motorists, in terms of driving or stopping in the cycle lane. The green and red lanes were consistently rated more positively than the blue lane, but whether green or red was preferred depended on whether the respondent had lived a place with coloured cycle lanes. People familiar with coloured cycle lanes, which in Norway are red, rated the red lane more positively than the green lane, while the opposite was true for people who were not used to coloured cycle lanes. The difference in ratings between different colours were similar to, or greater than, the difference from uncoloured to coloured, which implies that it not only matters *that* a lane is coloured, but also *which colour* it has.

## Introduction

Several countries around the world have started distinguishing cycle lanes by making part or all of them distinct in colour (e.g., New Zealand, United Kingdom, United States, Norway, Denmark, the Netherlands, etc.). Colour is intended to make the cycle lanes more visible and thus increase motorists’ awareness of cyclists and increase the predictability of cyclists (Brady et al., 2010; Høye et al., 2015). Differentiating the cycle lane further from the rest of the carriageway might also make it clearer that the cycle lane is an area reserved for cyclists and not for driving or parking (Høye et al., 2015), increase cyclists’ perceived safety, and promote cycling (The Norwegian Public Roads Administration [NPRA], 2019).

The use of coloured cycle lanes varies across countries, with some countries using coloured asphalt or paint on the full length of a cycle lane where motorists do not have access (e.g., Norway and the Netherlands) and others to highlight the presence of cyclists in the road without forbidding cars to drive on the coloured area (e.g., red “Cykelstrimmel” in Denmark). Others again mark intermittent sections rather than the full length (e.g., New Zealand) or use colour only to highlight zones where there is a higher risk for motorists-cyclist conflicts, for instance, in intersections (green in the US and blue in Denmark). For a detailed overview of cycling facilities in different countries, see Høye et al. (2015).

The colour used varies by country, with red being used in Norway, the Netherlands and Germany; green, in the US; no standard colour in England; and Denmark, both red and blue (Høye et al., 2015). There is a lack of consistency regarding the colour used and there appears to be a lack of discussion about, or justification for, why a certain colour is chosen in each area.

Colour research has indicated that different colours can evoke different emotional states in people and the Colour-in-Context theory emphasises the relationship between colour and affect, cognition, and behaviour (Elliot and Maier, 2012). The Colour-in-Context theory proposes that colours have immediate effects on psychological functioning when evaluating stimuli as hospitable or hostile, and can orient the person toward either approach-processes or avoidance-processes (Elliot and Maier, 2012) and that this impact can depend on the psychological context, for instance, that red can have an avoidance impact in intellectual contexts and an approach impact in romantic contexts (Meier et al., 2012).

If the colour itself contains information or invokes distinct emotions, for instance, that green is safe and red dangerous (Pravossoudovitch et al., 2014), then the effect of colouring a cycle lane could be diminished or amplified depending on the colour used. In psychological colour studies, red has been found to be the most arousing colour when controlling for brightness and saturation (Wilms and Oberfeld, 2018), and Stroop word evaluation tests have showed that categorisation of danger words and safety words was significantly faster when danger words were presented in red and safety words presented in green, indicating an implicit association between colour and safety/danger (Pravossoudovitch et al., 2014).

The Colour-in-Context theory states that the specific meanings of different colours result from both biological proclivities and social learning (Elliot and Maier, 2012). In some cases, these influences correspond with each other, for instance that red conveys danger both naturally (e.g., fire or blood) and is used to signal danger (e.g., stop signs and warning signals).

In the achievement context, red is often used to mark mistakes or corrections, and red has been found to have a negative impact in intelligence and achievement contexts (see overview in Elliot and Maier, 2012). However, opposite experiences with the colour red might reverse this association. In the Chinese stock market, red stands for an increase in stock price and green for a decrease, giving red a positive association. A study with Chinese students and Chinese stockbrokers found that while red (as opposed to green) had a negative impact on the students’ performance on an IQ test, the opposite effect was observed for stockbrokers (Zhang and Han, 2014).

While colour-specific effects might influence the ratings of the cycle lanes, one important aspect of this study is that Norway has increasingly been colouring cycle lanes red (The Norwegian Public Roads Administration [NPRA], 2019). Being familiar with red cycle lanes could influence the ratings of these just as much as any colour-specific associations.

The mere-exposure effect states that simply being familiar with a stimulus, such as sound or colour, will increase liking of it and a systematic review of two decades of research found that the exposure–affect relationship was robust and reliable (Bornstein, 1989). As there is no need to interact with the stimulus for exposure to increase liking (Bornstein, 1989), people would only need to have seen the red cycle lanes. A more recent meta-analysis of the mere-exposure effect (Montoya et al., 2017) found a consistent effect for visual stimuli. This effect could be characterised by an Inverted-U shaped curve, and the authors provide a new theoretical framework to account for the findings. Their Representation-Matching Model proposes that exposure to stimuli creates mental representations representing “how it should be” and that stimuli that match these mental representations are viewed as “good” and “correct,” which results in them being evaluated more favourably (Montoya et al., 2017).

If people in addition have positive experiences with red cycle lanes, they might not generalise those experiences to lanes of other colours, even if the effects would have been identical for any colour.

Looking at research into cycling infrastructure, field studies have consistently found that cyclists rate coloured cycle lanes as safer than uncoloured ones (Hunter et al., 2000; Jensen, 2006; Bjørnskau et al., 2016). As many view cycling as dangerous (e.g., Sener et al., 2009) and perceived risk of injury is an important barrier to cycling (e.g., Manaugh et al., 2017; Iwiñska et al., 2018), increases in perceived safety might lead to increased cycling, which is an important policy goal in Norway (The Norwegian Public Roads Administration [NPRA], 2012).

While the degree of cycling and cyclists’ perceived safety might be affected by the colour itself, it could also be influenced by changes in motorist behaviour. The effect of coloured cycle lanes on motorists’ behaviour has been examined for intersections (Hunter et al., 2000; Brady et al., 2010) and for stretches of road (City of New York Department of Transportation, 2011; LaMondia et al., 2019). The various studies have found that motorists kept a greater distance to cycle lanes after they were coloured (LaMondia et al., 2019) and reduced violations of the cycle lane, such as driving in it (City of New York Department of Transportation, 2011). In intersections, motorists were more likely to yield to cyclists after bicycle–motor vehicle crossings were coloured (Hunter et al., 2000; Brady et al., 2010).

While coloured cycle lanes have been evaluated, several of these are local reports and not peer-reviewed studies. Our search uncovered few peer-reviewed articles on the effects of coloured cycle lane and only one study which compared cycle lanes of different colours.

In Chile, coloured cycle lanes have been created with both different colours and different patterns (Vera-Villarroel et al., 2016). A survey was designed with pictures of three different patterns (full paint, chessboard-pattern or stripes) with the colour manipulated to five different colours (white, yellow, blue, red, or green), resulting in 15 different combinations. All pictures were then shown with the same three questions regarding perceived safety, salience, and liking. They found that colour explained more of the variance than pattern and that red was consistently given higher scores on the dependent variables (Vera-Villarroel et al., 2016). The three items used were all focussed on the colour (e.g., “the colour of this bike lane intersection seems appropriate for my safety”) and not on evaluations of the cycle lanes as a whole and if people ignored other aspects of the cycle lane this could have inflated any colour-specific effects.

There is an increasing amount of research into different kinds of cycle lanes and intersection solutions, such as bike boxes (Dill et al., 2012) or coloured lanes that highlight conflict areas (e.g., Hunter et al., 2000; Jensen, 2008; Brady et al., 2010). However, few studies examine the effect of specific features of these cycling facilities, such as the exact design, quality or location (Buehler and Dill, 2016). The present study isolates the effect of different colours to discover potential colour-specific effects that may have implications for the effectiveness of coloured cycling infrastructure.

As coloured cycle lanes are used more than they are evaluated, there is a dearth of evidence to justify using one colour over another. The aim of this study is to examine whether coloured cycle lanes are perceived differently, comparing different colours (blue, green, and red) to each other and to a line-delineated cycle lane with the same colour as the carriageway. The following research questions are explored, with no specific hypotheses.

RQ1: Do coloured cycle lanes differ from an uncoloured lane on perceived visibility, perceived safety for motorists and cyclists, being inviting to cycle in and estimated degrees of motorists’ violation of the cycle lane?

RQ2: Which colour is preferred?

RQ3: Do people with experience with coloured cycle lanes rate red cycle lanes as better than people without experience?

RQ4: Does it matter more to people that a cycle lane is coloured than which colour it is?

## Materials and Methods

The study was approved by the Norwegian Social Science Data Services, and all participants gave their informed consent before completing the questionnaire. The survey was created in and hosted by Quenchtec Survey Design and could be completed with all internet-compatible devices.

### Participants and Procedure

Respondents were recruited from a pool of participants from previous studies that had agreed to be contacted for future research. In August of 2019 they received an e-mail inviting them to respond to a short questionnaire. As the target group included both cyclists and motorists, the invitation did not specify that the survey focussed on coloured cycle lanes. Of the 1915 people invited to participate, 579 completed the survey and one person was excluded due to being partially colour blind.

The first questions related to frequency of cycling and driving. Depending on these answers, respondents were classified as either cyclists (cycled once a week or more often) or motorists and received mode-specific questions. These groups were used to filter the survey and ensure that people had experience with the mode they were asked about and were used for practical purposes, not to describe “who” the participants are. Some who were categorised as cyclists may have driven a car more often than they cycled, but their experience as a cyclist made them able to answer the cyclist-specific questions. People who didn’t drive at all and cycled less than weekly were excluded from the analyses (*n* = 18). Table 1 shows the sample characteristics.

**TABLE 1:** Sample characteristics.

	Cyclists (*n* = 206)		Motorists (*n* = 354)	
	*N*	*%*	*n*	*%*
*Men*	113	54.9	214	39.3
*Women*	92	44.7	139	60.5
*Other/don’t want to say*	1	0.5	1	0.3
*Lived place with coloured cycle lanes*	174	84.5	230	65
*Area of residence*				
*Oslo*	103	50	167	47.2
*Bergen*	30	14.6	86	24.3
*Trondheim*	52	25.2	62	17.5
*Stavanger*	18	8.7	38	10.7
*Other*	3	1.5	38	0.3
*Age category*				
*29 years or younger*	13	6.3	11	3.1
*30–49 years*	79	38.3	106	29.9
*50–69 years*	101	49	160	45.2
*70 years or older*	13	6.3	77	21.8

The invite e-mail included a link to the online survey. Each e-mail contained a unique link, and no respondent could answer more than once. The survey was designed with forced-choice, which ensured that we had practically no missing values; an error seems to have occurred as one respondent is missing a value on six items. As this only affected one respondent, there is no systematic trend for missing values.

### Items and Analyses

The relevant items from the survey are shown in the Appendix. Experience with coloured cycle lanes was operationalised as whether (yes or no) respondents said they have lived a place with coloured cycle lanes.

Respondents were asked to rate differently coloured cycle lanes. To avoid other differences, we used the same picture of a cycle lane and manipulated the colour with Photoshop. The original picture (Lode, 2015) showed an uncoloured cycle lane (Hue: 60°, Saturation: 5%, Brightness: 56%), which was changed to red (H: 12°, S: 19%, B: 60%), green (H: 106°, S: 19%, B: 60%), and blue (H: 194°, S: 19%, B: 60%). All colours were at low saturation rather than high saturation for two reasons: (1) coloured asphalt and paint fade over time and most cycle lanes will not sustain a high saturation and (2) a belief that showing pictures of highly saturated colours would increase the risk of a ceiling effect on the outcome variables (e.g., “very visible”).

Cyclists and motorists received somewhat different questions, but both groups rated the cycle lanes on four different items [visibility, safety (motorist or cyclist), how inviting it seemed to cycle (cyclists) and degree of motorists’ violation of the cycle lanes (motorist rated both other motorists and themselves while cyclists rated motorists in general)]. The uncoloured cycle lane was shown first for all items, but the order of red, green, and blue were randomised through a function of the survey programme. All items used seven-point scales.

We used two-way mixed ANOVAs to examine both the effect of different colours (within-subjects) and experience living with coloured cycle lanes (between-subjects). The analyses were conducted using IBM SPSS Statistics version 26.

Assumptions of outliers, normal residuals, homogeneity of variances, homogeneity of intercorrelations, and sphericity were checked for all mixed ANOVA analyses. Assumptions of homogeneity of variances, homogeneity of intercorrelations, and normality were met for all items. The sphericity assumption was violated in all cases and we therefore report the sphericity-corrected results, using either Huynh–Feldt or Greenhouse–Geisser correction depending on whether the epsilon was higher or lower than 0.75 (Field, 2018).

Two of the within-subject factors (motorists’ beliefs about their own degree of driving/stopping in the cycle lanes and visibility) had outliers with studentised residuals greater than ±3. Examination of the outliers showed that they are likely true unusual values, not measurement errors, and we were therefore loath to exclude them. To examine their impact on the results, we conducted the relevant ANOVAs (visibility and motorists’ own violation of cycle lane) both with and without the outliers and compared the results. For both analyses, the presence (or not) of the outliers had no meaningful impact on the results. While exact estimates and values were somewhat impacted (i.e., type III sum of squares were lower and *f*-values higher without outliers), there was no difference in whether main effect, interaction effect, simple contrasts, or pairwise comparisons were below or above the given alpha level of 0.05. As these differences don’t impact our interpretation of the results, and the outliers represent true values, we report the results with outliers.

As our interest was in which colours, if any, differed from the others, we conducted pairwise comparisons with Bonferroni corrections. Effect sizes are given as Cohen’s *d* and interpreted according to Cohen’s (1988) conventions. Cohen’s *d* is calculated for related samples and includes the correlation between measures. For items with a significant interaction, *post hoc* simple contrasts were carried out with the red cycle lane as the reference.

In addition to differences between colours, we wondered whether it mattered more that cycle lanes were coloured than which colour was chosen. All respondents were asked directly how important it was to them that the cycle lane had a different colour than the carriageway and how relevant it was to them whether that colour was red, green, or blue. We compared these ratings using a Wilcoxon signed-rank test.

In addition, we used ten Wilcoxon signed-rank tests to examine whether the differences from uncoloured to least positively rated coloured lane were greater or smaller than the differences between the least and the most positively rated coloured lane.

## Results

### Perceptions of Cycle Lane Characteristics

Respondents were asked to rate the differently coloured cycle lanes on several characteristics and research questions one, two, and three all focus on comparisons between the different colours on these characteristics. The following sections present the results from the mixed ANOVA for the items of visibility, cyclist safety, motorist safety, how inviting it seemed to cycle, and both cyclists’ and motorists’ assumptions of the degree to which motorists would violate the cycle lanes by driving or stopping in them.

#### Visibility

**FIGURE 1:**
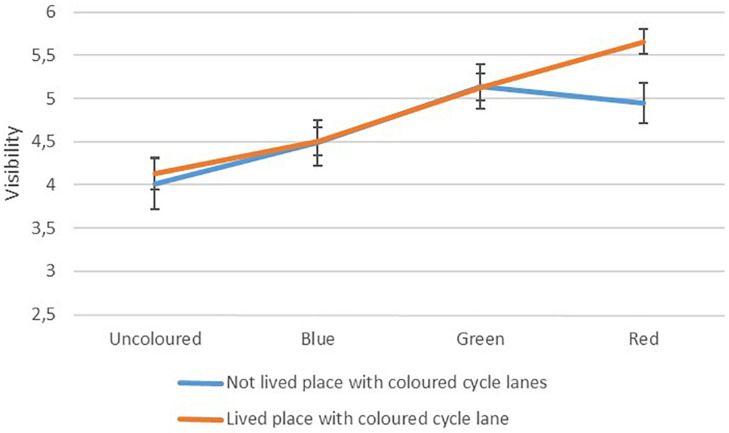
Visibility of cycle lanes of different colours, interacting with whether respondents have lived places with coloured cycle lanes, *n* = 560, graph scaled to show differences.

All respondents were asked to rate the visibility of the cycle lanes. Figure 1 shows the mean ratings, with 95% confidence intervals, for respondents with and without experience living in a city with coloured cycle lanes.

There was a significant main effect of colour on visibility ratings *F*(2.71,1511.7) = 100.288, *p* < 0.001 and a significant interaction between colour and experience living with coloured cycle lanes *F*(2.71,1511.7) = 8.96, *p* < 0.001. There was no significant main effect of experience.

The pairwise comparisons between different colours showed that the uncoloured and the blue cycle lanes were significantly different from each other and both the green and red lanes (*p* < 0.001 for all comparisons), while the difference between the green and the red cycle lane was not statistically significant (*p* = 0.212). Simple contrasts with the red cycle lane as reference showed significant interactions between colour and experience when comparing the red lane to the green lane *F*(1,558) = 21.56, *p* < 0.001, the blue lane *F*(1,558) = 20.73, *p* < 0.001 and the uncoloured lane *F*(1,558) = 10.08, *p* = 0.002.

The red lane was rated the most visible lane for the group that had lived a place with coloured cycle lanes, but not for the group that hadn’t. For the latter group, the green cycle lane had the highest rating on visibility. This did not support the idea that red is rated the most visible colour overall but did illustrate that people who were used to (red) coloured cycle lanes rated the red lane as more visible than people who were not used to coloured cycle lanes.

The difference in visibility ratings between the uncoloured lane and the red lane for people who had lived somewhere with coloured cycle lanes was 1.53 and represented a large effect (*d* = 0.79). For people who had not lived a place with coloured cycle lane, the difference was greatest between the uncoloured lane and the green lane (1.13) and this effect was medium (*d* = 0.58).

#### Cyclist Safety

**FIGURE 2:**
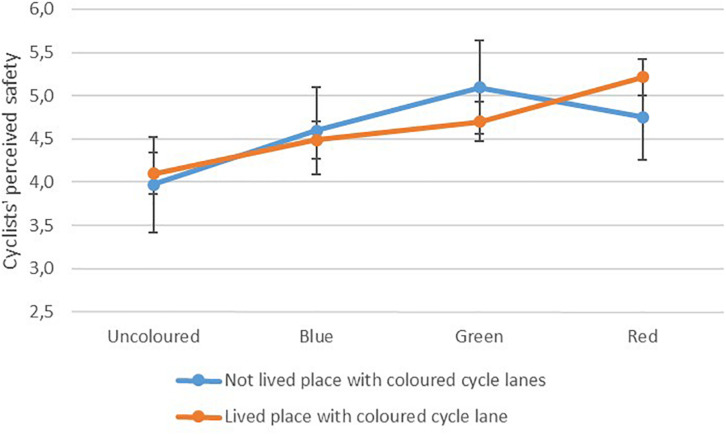
Cyclists’ safety evaluation and interaction with experience with coloured cycle lanes, *n* = 205, graph scaled to show differences.

Respondents categorised as cyclists were asked to rate how safe it seemed to cycle in each lane. Figure 2 shows the mean ratings, with 95% confidence intervals, for the different colours for respondents with and without experience living with coloured cycle lanes.

There was a significant main effect of colour *F*(2.73,553.45) = 22.09, *p* < 0.001 and a significant interaction between colour and experience living with coloured cycle lanes *F*(2.73,553.45) = 3.93, *p* = 0.011, but no significant main effect of experience. Pairwise comparisons between colours showed that the uncoloured cycle lane was significantly different from all the coloured lanes (*p* = 0.001 for the blue lane, *p* < 0.001 for the green and red) and the blue cycle lane was different from the green and red lanes (*p* = 0.002 for both).

The green and red lanes were not significantly different from each other for the main effect, but simple contrasts revealed that a significant interaction occurred when comparing the red lane with the green lane *F*(1,203) = 9.89, *p* = 0.002. In addition, there was a significant interaction between colour and experience when comparing the red lane with the blue lane *F*(1,203) = 5.56, *p* = 0.019.

For people who were used to coloured cycle lanes the red cycle lane was rated as the safest, while people who were not used to coloured lanes preferred the green lane. The difference in safety ratings between the uncoloured lane and the red lane for cyclists who had lived a place with coloured cycle lanes was 1.11 and represented a medium effect (*d* = 0.69). For people who had not lived a place with coloured cycle lane, the difference was greatest between the uncoloured lane and the green lane (1.12) and this effect was medium (*d* = 0.59).

#### Motorist Safety

**FIGURE 3:**
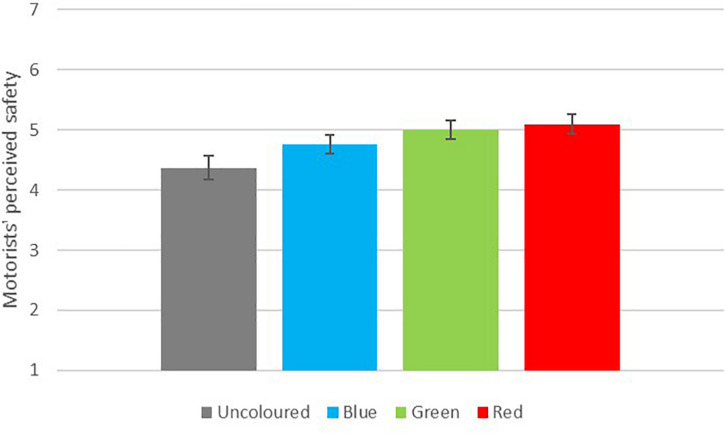
Motorists’ safety evaluation, *n* = 354.

Respondents categorised as motorists were asked how safe it seemed to drive in the road next to each coloured cycle lane. There was no statistically significant interaction between colour and experience for motorists’ safety ratings and no significant main effect of experience. Figure 3 shows the mean ratings of motorist safety for each pictured cycle lane.

There was a significant main effect of colour on motorists’ safety evaluations *F*(2.28,800.74) = 36.54, *p* < 0.001. Pairwise comparisons showed that the uncoloured cycle lane and the blue cycle lane were significantly different both from each other and from the green and red lanes (*p* < 0.001 for all comparisons). While the red lane had a slightly higher average rating of safety than the green lane, this difference was not statistically significant (*p* = 0.781). The difference between motorists’ safety ratings of the uncoloured lane and the red lane was 0.76 and represented a small to medium effect (*d* = 0.45).

#### Inviting to Cycle

**FIGURE 4:**
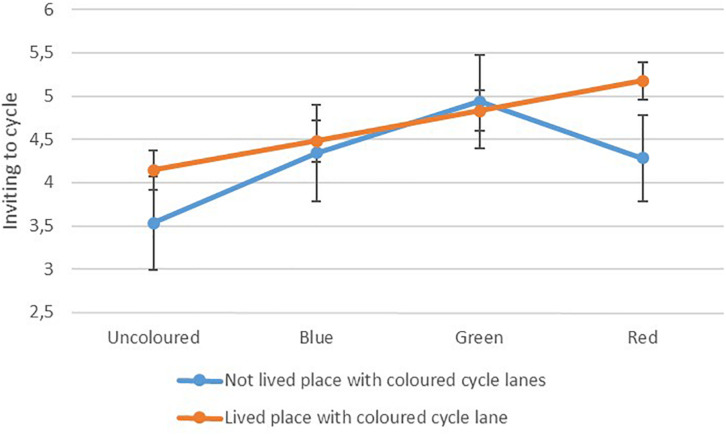
Cyclists’ rating of how inviting it seems to cycle in lanes of different colours, interacting with whether they have experience with coloured cycle lanes, *n* = 205, graph scaled to show differences.

Cyclists were asked how inviting it seemed to cycle in each cycle lane and Figure 4 shows the mean ratings with 95% confidence intervals.

There was a main effect of colour *F*(2.77,563.72) = 21.47, *p* < 0.001 and an interaction between colour and having lived a place with coloured cycle lanes *F*(2.77,563.72) = 5.16, *p* = 0.002. There was no significant main effect of experience. Of the pairwise comparisons between colours there were significant differences between the uncoloured cycle lane and all the coloured lanes and between the blue lane and the green lane (*p* < 0.001 for all comparisons).

When looking at the main effect of colour without the interaction, the red cycle lane was not significantly different from either the blue lane or the green lane. However, as illustrated by Figure 4, there was a significant interaction between colour and experience living with coloured cycle lanes. This interaction was significant for simple contrasts between the red lane and both the blue lane *F*(1,203) = 6.05, *p* = 0.015 and the green lane *F*(1,203) = 10.98, *p* = 0.001. Those unused to coloured cycle lanes rated the green lane as the most inviting and respondents with experience rated the red lane as most inviting. For people who had lived a place with coloured cycle lanes, the mean difference in ratings between how inviting it seemed to cycle in the red cycle lane versus the uncoloured was 1.04 and represented a medium to large effect size (*d* = 0.73). People who had not lived a place with coloured cycle lanes rated the green lane as most inviting and the difference in ratings of the green lane and the uncoloured lane was 1.41 and represented a medium effect size (*d* = 0.79).

#### Motorists Driving/Stopping in Cycle Lanes

**FIGURE 5:**
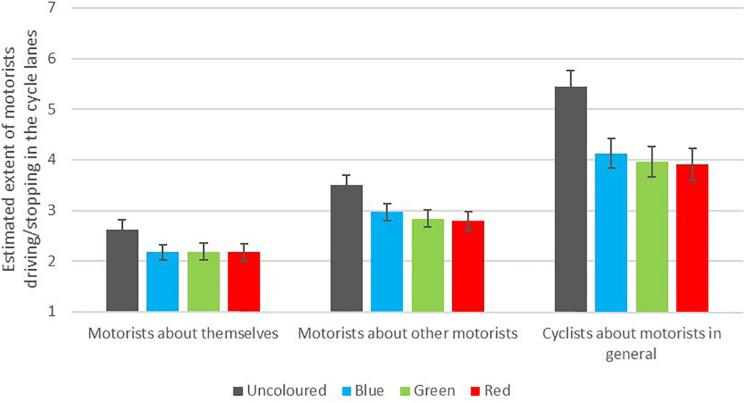
Beliefs about motorists’ tendency to drive/stop in cycle lane for cyclists and motorists, *n* = 354 for motorist items and *n* = 206 for cyclist item.

Figure 5 shows how cyclists and motorists assessed the likelihood of motorists stopping in the cycle lane. In general, red and green were rated as the most deterring colours, followed by blue.

There was no significant interaction between colour and having lived a place with coloured cycle lanes for any of the three items, nor was there any significant main effect of experience. The main effect of colour, however, was significant for cyclists’ estimates of motorists degree of driving/stopping in the cycle lanes, *F*(2.16,439.98) = 47.93, *p* < 0.001, for motorists’ estimates of other motorists’ degree of violating the cycle lanes, *F*(2.01,707.91) = 36.22, *p* < 0.001, and for motorists’ beliefs about the extent they themselves would drive/stop in the cycle lanes pictured, *F*(2.02,709.51) = 22.39, *p* < 0.001.

Pairwise comparisons showed that the differences between uncoloured and all the coloured lanes were statistically significant in all three cases, whereas the differences between green and the red cycle lanes were not statistically significant for any of the questions. For motorists’ ratings of other motorists, there were statistically significant differences between ratings of the blue lane and both the green lane (*p* = 0.025) and the red lane (*p* = 0.028). The blue lane was not significantly different from either the green lane (*p* = 0.628) or the red lane (*p* = 0.698) when cyclists rated motorists in general, nor when motorists rated their own degree of violating the cycle lane (*p* = 1.000 for both comparisons).

The greatest difference between colours was between the uncoloured lane and the red lane when rated by cyclists, with a medium to large effect size of *d* = 0.76. The smallest statistically significant difference was motorists’ estimates of other motorists’ tendency to drive/stop in the blue versus the green lane, with a marginal to small effect size (*d* = 0.15).

Overall, cyclists believed that motorists would drive/stop in the lanes to a higher degree than motorists believed regarding both ‘other motorists’ and themselves. Motorists thought it was more likely that other car drivers would stop in the cycle lane than they would themselves.

#### Does Colouring Have an Effect, and Which Colour Is Best?

Our first research question was whether the coloured cycle lanes differed from the uncoloured lane on the given characteristics. Overall, all items showed a significant main effect of colour and the pairwise comparisons showed that the ratings of the uncoloured cycle lane differed significantly from ratings of all other colours on all items. While the exact magnitude of the difference varied both depending on colour and on characteristic rated, coloured cycle lanes were consistently rated as more visible, safer, more inviting to cycle, in and more deterring to motorists.

Research questions two and three related to whether one colour was preferred over others and whether experience with coloured cycle lane would influence the preferred colour, respectively. We found an overall trend in the order of colours rated least to most positive, with the uncoloured lane being rated lowest and either the green or red lane rated highest. There was a significant interaction between colour and experience on three of the seven items, with no interaction on the items relating to motorists specifically (motorists’ perceived safety and items about motorists violating the cycle lane). Where there was an interaction between colour and experience, green was the preferred colour for those without experience with coloured cycle lanes and red the preferred colour for those with experience. For items with no significant interaction, there was also no statistically significant difference between the ratings of the green and red cycle lanes.

### Importance and Relevance of Colour

When asked directly about the importance of the cycle lane being coloured and the relevance of which colour is used, respondents gave a higher score to importance (*Mdn* = 5) than to relevance (*Mdn* = 4). A Wilcoxon signed-rank test showed that this difference was statistically significant, *z* = 11.015, *p* < 0.001.

By looking at the ratings of the different colours, and the differences between them, we can examine whether this stated belief translated to actual scores. The effect of colouring a lane was defined as the difference between the uncoloured lane and the least positively rated coloured lane and the effect of different colours was defined as the difference between the least and the most positively rated coloured lane.

As the interaction with having lived a place with coloured cycle lanes influenced which colour was rated most positively, the comparisons were conducted separately for people with/without experience. In total, ten sets of differences were compared and Table 2 shows the results.

**TABLE 2:** Wilcoxon signed-rank tests of effect of colouring minus effect between colours.

	Positive differences	Negative differences	Ties	z	*p*
Visibility *(with experience)*	101	205	98	–5.813	< 0.001
Visibility *(without experience)*	63	56	37	–0.535	0.593
Cyclist safety *(with experience)*	49	66	58	–2.153	0.031
Cyclist safety *(without experience)*	11	8	13	0.592	0.554
Inviting to cycle *(with experience)*	53	64	56	–1.657	0.097
Inviting to cycle *(without experience)*	9	10	13	–0.061	0.951
Motorist safety	95	101	158	–0.009	0.993
Cyclists’ beliefs about motorists’ violation of the cycle lanes	96	41	69	4.466	< 0.001
Motorists’ beliefs about other motorists’ violation	115	70	169	3.646	< 0.001
Motorists’ beliefs about own violation	100	45	209	4.551	< 0.001

If colouring the cycle lane was more important than which colour was used, we would expect the effect of colour to consistently be bigger than the effect of different colours. Out of the 10 comparisons, only five had a statistically significant difference between the two effects. As shown in Table 2, three of the five significant differences went in the direction of colouring at all having a greater effect than which colour was used, while the remaining two went in the opposite direction. For respondents who had lived a place with coloured cycle lanes, the ratings of cyclist safety and visibility of the cycle lane were more affected by the difference between the least and most positively rated colours (blue and red, respectively) than between the uncoloured lane and the least positively rated coloured lane. For the other comparisons, there were no statistically significant differences in the effect of colouring versus the effect between different colours.

## Discussion

### Is Red the Best Colour?

As red is the chosen colour for cycle lanes in Norway we wondered whether red would be rated as the most preferred colour. However, our results don’t enable us to conclude that either red, green or blue is *the best* overall colour to use for a cycle lane.

The magnitude of the differences between colours varied between items and, in some cases, depending on whether respondents were or were not used to coloured cycle lanes. There was, however, an overall trend in the order of colours rated least to most positive; the uncoloured cycle lane was consistently rated least positive, the blue lane slightly more positive and the green and red lane were usually rated most positive. The differences between the green and red lanes were either not significant or depended on experience with coloured cycle lanes. The findings do indicate, however, that green and red cycle lanes are preferred over blue and uncoloured lanes.

As the colour red has been found to be associated with danger, we expected that the red lane would be most deterring to motorists. There were no statistically significant differences between the green and red lanes for any of the items and this assumption that the red lane would be more deterring was not supported for estimations of motorists’ tendency to drive/stop in cycle lanes. When motorists rated other motorist behaviour, however, there was a statistically significant difference between ratings of the blue cycle lane and both the red and green lanes, similar to that found for the other cycle lane characteristics. This indicates that the different ratings were not due to a warning element inherent in the colour red, but rather something else that is shared between the green and red cycle lanes that sets them apart from the blue lane.

One of the justifications for cycle lane colour choice in the US it that it is not permitted to use the colour blue for cycle lanes as blue is the primary colour of the international symbol of accessibility parking (Lindley, 2011). Respondents in Norway might also have been influenced by the association between blue asphalt and parking and thus rated the blue cycle lane less as less deterring to motorists than the green or red lanes, as the latter colours are not generally associated with other transport modes.

### Effects of Having Lived a Place With (Red) Coloured Cycle Lanes

Of the four largest cities in Norway, all except one had some presence of red cycle lanes at the time of the survey (The Norwegian Public Roads Administration [NPRA], 2019) and a majority of respondents had therefore lived a place with coloured cycle lanes.

When comparing only the main effect of colour there were no significant differences between ratings of the green and red cycle lane on any items. Three items, however, had a significant interaction between colour and whether respondents had lived a place with coloured cycle lanes or not. People who were familiar with (red) coloured cycle lanes rated the red lane as the safest, most visible and most inviting lane, whereas people who had not lived a place with coloured cycle lanes rated the green lane most positively on all those items. That people who were familiar with red cycle lane rated the red lane as the most inviting indicates that even if the red colour originally is more deterring than other colours, cyclists who are used to red cycle lanes will find them more inviting than differently coloured cycle lanes.

We expected that people who had lived a place with coloured cycle lanes would rate the red lane more positively than people who had not. This study doesn’t reveal why this preference occurs, but possible explanations include such respondents having previous positive experiences with red cycle lanes (Zhang and Han, 2014) and that being exposed to red cycle lanes increases liking (Bornstein, 1989; Montoya et al., 2017).

As Norway only uses red cycle lanes we couldn’t examine whether this increased preference for the familiarly coloured cycle lane would also occur for green and blue coloured cycle lanes, or whether blue cycle lanes would continue to be rated less positively than red or green lanes. Furthermore, as our “experience” variable was binary, we could not quantify exposure and therefore weren’t able to examine whether there was an inverted U-shaped effect of experience, like that found by Montoya et al. (2017) in their review of the mere-exposure effect.

### More Important That a Cycle Lane Is Coloured Than Which Coloured Is Used?

A common reason for colouring cycle lanes is to make them more visible and increase motorists’ awareness of both cyclists and their section of the road. Arguably, any colour that differentiates the lane from the rest of the carriageway could achieve this and any differences between colours could seem insignificant compared to the effect of using any colour at all. In this study we found that all coloured cycle lanes were rated significantly more positively than the uncoloured lane, on all items. These findings support the general effort of colouring cycle lanes.

However, the fact that any colour is an improvement to no colour does not mean that “all colours are created equal.” There were significant differences between colours for nearly all items. The only two items where there were no significant differences between colours were motorists’ rating of their own tendency to drive/stop in the cycle lanes and cyclists’ ratings of motorists’ tendency to do the same. For the former, the mean ratings for all colours were very low (just below 2.2) indicating that the question might have suffered from a floor effect.

When asked directly, respondents thought colouring at all was more important than which colour used, but did not think colour choice was irrelevant. To see if this translated to actual ratings, we compared the magnitudes of the differences to see whether the greatest effect was from the uncoloured lane to the least positively rated coloured lane, or from the least to the most positively rated coloured lane. If colouring at all was more important than which colour used, we would expect these differences to be in favour of the effect of colour being greater than the effect of different colours.

For half of the comparisons, there was no significant difference between the effect of colour and the effect of different colours and for the other five, three went in the direction of colouring at all having the greatest effect, while two items had a greater difference between colours. Rather than colouring being more important, this indicates that choice of colour might be just as important as colouring at all. While our respondents, on average, said that colouring was more important than the choice of colour, the actual ratings revealed that this varied depending on the characteristic considered.

Colouring a cycle lane seems a worthwhile effort regardless of which colour is used, but our results illustrate the importance of considering choice of colour wisely. The clear trend of the blue lane being rated less positively than the green or red lanes indicates that the benefits of colouring cycle lanes could be enhanced or diminished depending on the colour.

### Strengths and Limitations

Our sample was relatively large (*N* = 560) and consisted of approximately equal shares of men and women, a broad age distribution, and people from four different cities in Norway. We had also respondents who had and had not lived a place with coloured cycle lanes, a factor that was revealed to be important for the ratings of cycle lanes.

However, there are characteristics we did not measure and that are likely less representative due to both our own sampling criteria (we needed a certain amount of cyclists) and self-selection to the survey. Respondents were recruited through previous participants in studies conducted by the Institute of Transport Economics who had agreed to be contacted for future research. Almost 70% of those invited did not respond to the survey. This means that those who completed the survey had gone through three levels of “saying yes”; to the first study they participated in, to be contacted again at a later time, and to this specific survey. It is likely that they are somewhat different from people who said no at any one of those time points.

While psychological colour research has been a popular topic over decades, research into the affective effects of colour has suffered from methodological problems and results have been somewhat inconsistent (Elliot and Maier, 2012; Wilms and Oberfeld, 2018). In particular, few studies have controlled for saturation and brightness when comparing colours, meaning that results for example could be due to the prototypical colour red being relatively dark and saturated, and not the specific hue that makes a colour red (Pravossoudovitch et al., 2014).

As our interest was only in hue, we controlled for saturation and brightness. The same picture was used, with colours manipulated, and there was nothing else changing around the cycle lane that could impact differences in ratings. As participants answered on their own devices we could not control their screens and settings, but we expect that such differences would have impacted all colours the same and not impacted the differences between colours.

The presentation of the colours was randomised to counter any potential order-effect, but the uncoloured lane was always presented first. This could have exacerbated the observed differences between the uncoloured lane and all colours, though respondents were shown all pictures together before the first question.

If the colour red elicits a sense of warning or forbidding, and the colour green a sense of safety and welcoming, these effects are not necessarily part of our conscious awareness. The nature of self-report is that people need to be aware of something to report it. Asking more general questions (safety, visibility, inviting) we aimed to capture any subconscious effects of colour, but we cannot be sure that any differences between colour is due to the colours’ danger or safety association rather than any other, unmeasured, difference between the colours.

In addition to asking respondents about their own perceptions of the differently coloured cycle lanes, we asked them to estimate motorists’ tendency to drive/stop in the cycle lane. That is, we asked them to estimate others’ and their own partially hypothetical (for the unfamiliarly coloured lanes) behaviour. The marked differences between cyclists’ estimation of motorist behaviour and motorists’ estimation of both other motorists’ behaviour and their own behaviour illustrate why this does not translate to an objective measurement of driving/stopping in cycle lanes. However, the within-subject design means that any biases should be the same for all colours. It doesn’t matter whether cyclists or motorists were “more correct” when estimating motorists’ tendency to drive/stop in the cycle lanes, as we weren’t looking at the total degree, but whether people believed different coloured cycle lanes would increase/decrease the extent of motorist violation.

A potential confounding factor is that the pictured cycle lane is a real cycle lane in downtown Oslo, and any respondents who recognised the street might have answered the questions based on their knowledge of that particular street rather than as a random street. However, several researchers who are familiar with the street did not recognise it as the picture did not contain any unique identifiers and seemed a generic street with store fronts. In addition, familiarity with the street should only impact the total scores and not the difference between colours. While some respondents may have recognised the street we consider it unlikely to have had much of an impact on the results.

To operationalise “experience” we asked respondents whether they have lived a place with coloured cycle lanes. Ideally, we should instead have asked directly whether they have experience with cycling/driving in areas with coloured cycle lanes, and if so *which* colour. While Norway only uses red cycle lanes, some of the respondent may have lived abroad and become familiar with other colours.

One limitation to the study is that we did not include a question of colour vision, meaning we don’t know if the respondents were colour blind. However, the survey ended with a field for open comments and one respondent replied that they were “partially colour blind” and was therefore removed from the analyses. We believe that other colour blind people either would have not completed the survey or left a similar comment.

The one study we found that had previously looked at differently coloured cycle lanes did not include colour psychology or theory. A strength of the current study is that it attempts to bridge the gap between psychological theory and applied knowledge of infrastructural design for road users, providing a theoretical background for the observed effects.

### Policy Implications

The results from this web survey do not justify an effort to switch from red coloured cycle lanes in Norway, even if green lanes are rated more positively by people who haven’t lived a place with coloured cycle lanes. Being exposed to, and familiar with, red cycle lanes seem to result in overall more positive estimations of red lanes.

Overall, both motorists’ and cyclists’ ratings differed from uncoloured to coloured, with more positive ratings of the coloured lanes (specifically green and red). These results are consistent with field experiments showing increased perceived safety of cycle lanes after they were coloured (Hunter et al., 2000; Bjørnskau et al., 2016).

A combination of a cycle lane being rated as safer and more inviting can be assumed to lead to an increase in cycling and previous research has found that coloured cycle lane experience a greater increase in cycling than the overall increase in Oslo (Fyhri et al., 2019). Our findings therefore support existing evidence in favour of colouring cycle lanes. It should be emphasised that the results do not support building cycle lanes (where cyclists are placed level to the cars) instead of separate (protected) cycling infrastructure. Rather, they suggest that in a situation where separated cycle infrastructure is not an option, using a colour (preferably red or green) to make the lane more salient is beneficial.

### Future Research

More research is needed to examine whether similar patterns are found in other populations, especially countries with different cycling cultures and countries using other colours, or not using coloured cycle lanes at all (yet). It would be especially interesting to see whether experience with blue cycle lanes would make the blue lanes the most positively rated, or whether green or red lanes would still be preferred.

Experience with coloured cycle lanes depends on exposure in real-life and can be difficult to quantify. Even so, it would be an interesting point for future research to examine whether the effect of experience varies with degree of exposure (e.g., how often respondents have seen/used the coloured cycle lane, or perhaps how recent the colouring is in their area).

In addition to similar surveys in different countries, field studies should ideally be conducted with objective measurements of both motorist and cyclist behaviour, as well as survey data, before and after cycle lanes of different colours are implemented. As most countries choose one specific colour it might be necessary to compare results across nations.

## Conclusion

The results from this study contributes to understanding the effects of different colours used on cycle lanes and the impact of colour choice on important cycle lane characteristics.

For almost all variables examined there was a clear preference for either the green cycle lane or the red cycle lane. Blue was consistently the least positively rated colour, both for people who had lived a place with coloured cycle lanes and for people that hadn’t. Based on these results we therefore cannot recommend colouring cycle lanes blue. We don’t know, however, how blue would be rated in other countries, especially countries where blue is used as the standard colour for cycle lanes.

## Data Availability Statement

The raw data supporting the conclusions of this article will be made available by the authors, without undue reservation.

## Ethics Statement

The studies involving human participants were reviewed and approved by Norwegian Centre for Research Data, a national committee. The patients/participants provided their written informed consent to participate in this study.

## Author Contributions

KK conceived the idea and the experimental design of the study. AF was the project leader and responsible for project planning and funding, as well as ethical approval, and edited the manuscript. KK and AF together discussed and planned the specific survey experiment, which was designed by KK and distributed by AF. KK did the data analyses mainly with assistance from AF. KK and AF interpreted the results. KK wrote the manuscript. Both authors contributed to the article and approved the submitted version.

## Conflict of Interest

The authors declare that the research was conducted in the absence of any commercial or financial relationships that could be construed as a potential conflict of interest.
