# Supplementary material for: Is Red the New Black? A Quasi-Experimental Study Comparing Perceptions of Differently Coloured Cycle Lanes
Source: Front Psychol. 2020 Dec 4;11:554488. doi: 10.3389/fpsyg.2020.554488 (PMC7746616; doi:10.3389/fpsyg.2020.554488)
Supplement: Supplementary file 1 [file Table_1.docx]

**Survey questions translated**

**How often do you cycle at this time of year?**

5 days a week or more

3 to 4 days a week

1 to 2 days a week

1 to 3 days a month

Less often

**How often do you drive a car at this time of year?**

5 days a week or more

3 to 4 days a week

1 to 2 days a week

1 to 3 days a month

Less often

I don’t drive

**Information page**

In this survey, you’ll be shown pictures of cycle lanes. We sometimes ask you to evaluate the cycle lane as a whole and sometimes only the colour.

These are the pictures you will be asked about.


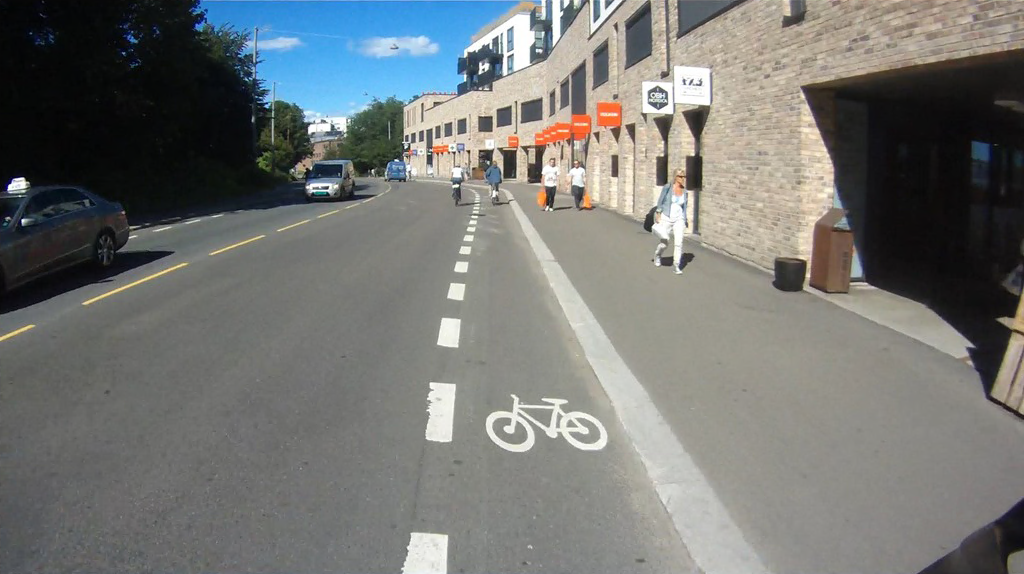


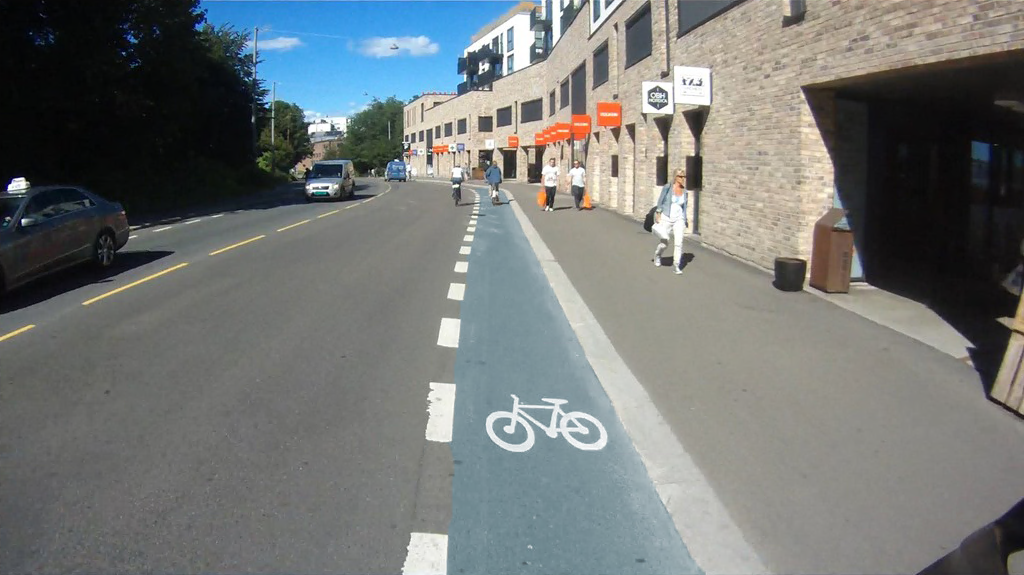


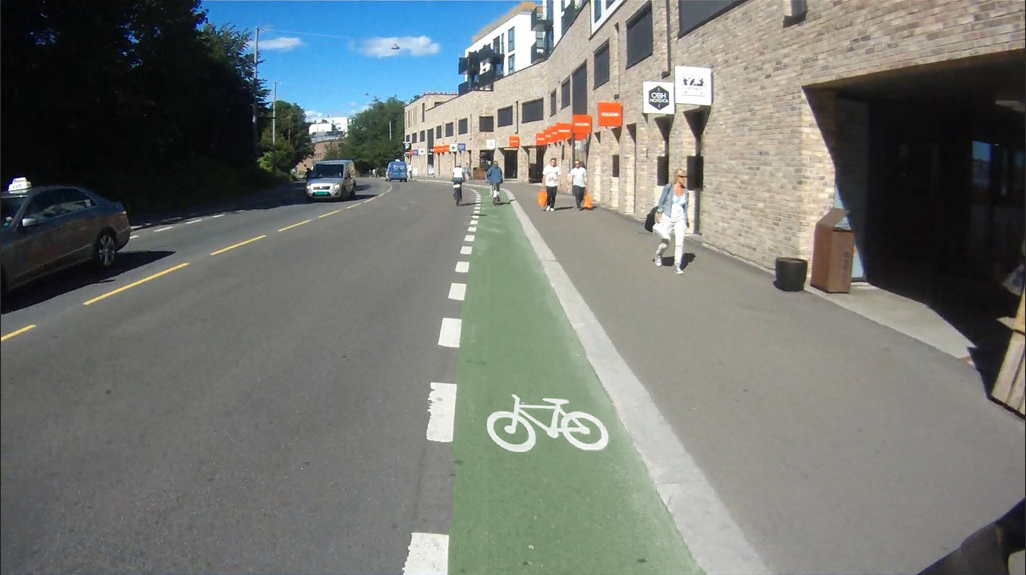


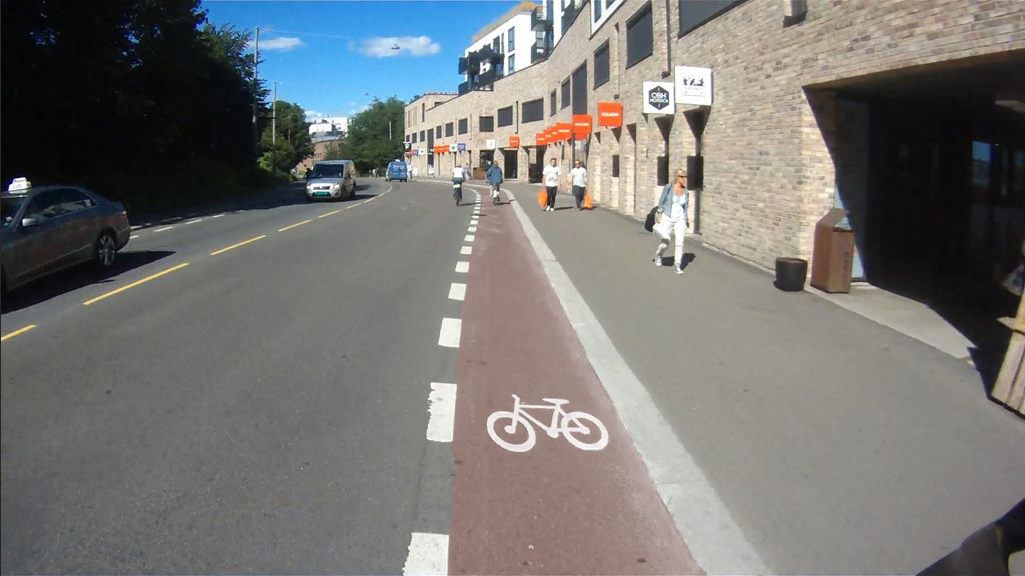


© Original uncoloured cycle lane picture by Lode, M. H. (2015) <https://mortenhowlode.wordpress.com/2015/08/14/hvor-kan-man-da-sykle-om-ikke-midt-i-veien/>

[All respondents] **To me, this cycle lane is …**

[Picture with uncoloured lane shown first, then randomized order of colour]

1 Barely visible

2

3

4

5

6

7 Very visible

[Cyclists] **To me, to cycle in this cycle lane seems …**

[Picture with uncoloured lane shown first, then randomized order of colour]

1 Very uninviting

2

3

4

5

6

7 Very inviting

[Cyclists] **To me, cycling in this cycle lane seems …**

[Picture with uncoloured lane shown first, then randomized order of colour]

1 Very unsafe

2

3

4

5

6

7 Very safe

[Motorists] **To me, driving a car in this street seems …**

[Picture with uncoloured lane shown first, then randomized order of colour]

1 Very unsafe

2

3

4

5

6

7 Very safe

[Cyclists] **To what extent do you think motorists will drive or stop in the bike lane pictured?**

[Picture with uncoloured lane shown first, then randomized order of colour]

1 To a very small extent

2

3

4

5

6

7 To a very large extent

[Motorists] **To what extent do you imagine you could drive or stop in the cycle lane pictured**?

[Picture with uncoloured lane shown first, then randomized order of colour]

1 To a very small extent

2

3

4

5

6

7 To a very large extent

[Motorists] **To what extent do you think motorists in general will drive or stop in the bike lane pictured?**

[Picture with uncoloured lane shown first, then randomized order of colour]

1 To a very small

2

3

4

5

6

7 To a very large extent

[All] **To me, that the cycle lane has a different colour than the carriageway is …**

1 Very unimportant

2

3

4

5

6

7 Very important

[All] **To me, whether the cycle lane is red, green or blue is …**

1 Very irrelevant

2

3

4

5

6

7 Very relevant

[All] **Where do you live now?**

Oslo and surrounding area

Bergen and surrounding area

Trondheim and surrounding area

Stavanger and surrounding area

Other

[All] **Have you lived a place with coloured cycle lanes?**

Yes

No

[All] **How old are you?**

Under 20

20-29

30-39

40-49

50-59

60-69

70-79

80 or older

[All] **Finally, we’d like to know your gender**

Man

Woman

Other/don’t want to say

[All] **Do you have any comments to the survey?**

Please do not provide health information in the comments section.

Write here:
