# Supplementary material for: Is Red the New Black? A Quasi-Experimental Study Comparing Perceptions of Differently Coloured Cycle Lanes
Source: Front Psychol. 2020 Dec 4;11:554488. doi: 10.3389/fpsyg.2020.554488 (PMC7746616; doi:10.3389/fpsyg.2020.554488)
Supplement: Supplementary file 2 [file Table_2.DOCX]

Table 1. Pairwise comparisons of visibility ratings

| (I) colour | (J) colour | Mean Difference (I-J)^a^ | Std. Error | Sig.^b^ | 95% Confidence Interval for Difference^b^ | |
| --- | --- | --- | --- | --- | --- | --- |
|  |  |  |  |  | Lower Bound | Upper Bound |
| 1 Uncoloured | 2 | -.429^*^ | .078 | .000 | -.635 | -.222 |
|  | 3 | -1.069^*^ | .090 | .000 | -1.307 | -.830 |
|  | 4 | -1.234^*^ | .094 | .000 | -1.483 | -.985 |
| 2 Blue | 1 | .429^*^ | .078 | .000 | .222 | .635 |
|  | 3 | -.640^*^ | .065 | .000 | -.811 | -.469 |
|  | 4 | -.806^*^ | .077 | .000 | -1.009 | -.602 |
| 3 Green | 1 | 1.069^*^ | .090 | .000 | .830 | 1.307 |
|  | 2 | .640^*^ | .065 | .000 | .469 | .811 |
|  | 4 | -.165 | .078 | .212 | -.373 | .042 |
| 4 Red | 1 | 1.234^*^ | .094 | .000 | .985 | 1.483 |
|  | 2 | .806^*^ | .077 | .000 | .602 | 1.009 |
|  | 3 | .165 | .078 | .212 | -.042 | .373 |
|  | | | | | | |

Note. ^a^Based on estimated marginal means, ^b^Adjustment for multiple comparisons: Bonferroni. * The mean difference is significant at the .05 level.

Table 2. Pairwise comparisons of cyclists’ perceived safety ratings

| (I) colour | (J) colour | Mean Difference (I-J)^a^ | Std. Error | Sig.^b^ | 95% Confidence Interval for Difference^b^ | |
| --- | --- | --- | --- | --- | --- | --- |
|  |  |  |  |  | Lower Bound | Upper Bound |
| 1 Uncoloured | 2 | -.506^*^ | .128 | .001 | -.848 | -.164 |
|  | 3 | -.863^*^ | .136 | .000 | -1.225 | -.501 |
|  | 4 | -.948^*^ | .152 | .000 | -1.354 | -.543 |
| 2 Blue | 1 | .506^*^ | .128 | .001 | .164 | .848 |
|  | 3 | -.357^*^ | .098 | .002 | -.618 | -.096 |
|  | 4 | -.442^*^ | .121 | .002 | -.765 | -.119 |
| 3 Green | 1 | .863^*^ | .136 | .000 | .501 | 1.225 |
|  | 2 | .357^*^ | .098 | .002 | .096 | .618 |
|  | 4 | -.085 | .136 | 1.000 | -.449 | .278 |
| 4 Red | 1 | .948^*^ | .152 | .000 | .543 | 1.354 |
|  | 2 | .442^*^ | .121 | .002 | .119 | .765 |
|  | 3 | .085 | .136 | 1.000 | -.278 | .449 |

Note. ^a^Based on estimated marginal means, ^b^Adjustment for multiple comparisons: Bonferroni. * The mean difference is significant at the .05 level.

Table 3. Pairwise comparisons of how inviting it seems to cycle in the cycle lanes pictured

| (I) colour | (J) colour | Mean Difference (I-J)^a^ | Std. Error | Sig.^b^ | 95% Confidence Interval for Difference^b^ | |
| --- | --- | --- | --- | --- | --- | --- |
|  |  |  |  |  | Lower Bound | Upper Bound |
| 1 Uncoloured | 2 | -.574^*^ | .138 | .000 | -.941 | -.207 |
|  | 3 | -1.047^*^ | .143 | .000 | -1.428 | -.666 |
|  | 4 | -.892^*^ | .146 | .000 | -1.281 | -.504 |
| 2 Blue | 1 | .574^*^ | .138 | .000 | .207 | .941 |
|  | 3 | -.473^*^ | .110 | .000 | -.767 | -.179 |
|  | 4 | -.318 | .155 | .247 | -.731 | .094 |
| 3 Green | 1 | 1.047^*^ | .143 | .000 | .666 | 1.428 |
|  | 2 | .473^*^ | .110 | .000 | .179 | .767 |
|  | 4 | .155 | .151 | 1.000 | -.249 | .558 |
| 4 Red | 1 | .892^*^ | .146 | .000 | .504 | 1.281 |
|  | 2 | .318 | .155 | .247 | -.094 | .731 |
|  | 3 | -.155 | .151 | 1.000 | -.558 | .249 |

Note. ^a^Based on estimated marginal means, ^b^Adjustment for multiple comparisons: Bonferroni. * The mean difference is significant at the .05 level.

Table 4. Pairwise comparisons of motorists’ perceived safety ratings

| (I) colour | (J) colour | Mean Difference (I-J)^a^ | Std. Error | Sig.^b^ | 95% Confidence Interval for Difference^b^ | |
| --- | --- | --- | --- | --- | --- | --- |
|  |  |  |  |  | Lower Bound | Upper Bound |
| 1 Uncoloured | 2 | -.391^*^ | .083 | .000 | -.610 | -.172 |
|  | 3 | -.631^*^ | .090 | .000 | -.870 | -.391 |
|  | 4 | -.728^*^ | .094 | .000 | -.978 | -.478 |
| 2 Blue | 1 | .391^*^ | .083 | .000 | .172 | .610 |
|  | 3 | -.240^*^ | .051 | .000 | -.376 | -.104 |
|  | 4 | -.337^*^ | .062 | .000 | -.503 | -.171 |
| 3 Green | 1 | .631^*^ | .090 | .000 | .391 | .870 |
|  | 2 | .240^*^ | .051 | .000 | .104 | .376 |
|  | 4 | -.097 | .064 | .781 | -.267 | .073 |
| 4 Red | 1 | .728^*^ | .094 | .000 | .478 | .978 |
|  | 2 | .337^*^ | .062 | .000 | .171 | .503 |
|  | 3 | .097 | .064 | .781 | -.073 | .267 |

Note. ^a^Based on estimated marginal means, ^b^Adjustment for multiple comparisons: Bonferroni. * The mean difference is significant at the .05 level.

Table 5. Pairwise comparisons of motorists’ estimate of their own tendency to drive/stop in the cycle lanes pictured

| (I) colour | (J) colour | Mean Difference (I-J)^a^ | Std. Error | Sig.^b^ | 95% Confidence Interval for Difference^b^ | |
| --- | --- | --- | --- | --- | --- | --- |
|  |  |  |  |  | Lower Bound | Upper Bound |
| 1 Uncoloured | 2 | .446^*^ | .072 | .000 | .256 | .636 |
|  | 3 | .433^*^ | .081 | .000 | .219 | .647 |
|  | 4 | .446^*^ | .086 | .000 | .218 | .675 |
| 2 Blue | 1 | -.446^*^ | .072 | .000 | -.636 | -.256 |
|  | 3 | -.013 | .039 | 1.000 | -.117 | .092 |
|  | 4 | 7.013E-5 | .051 | 1.000 | -.134 | .135 |
| 3 Green | 1 | -.433^*^ | .081 | .000 | -.647 | -.219 |
|  | 2 | .013 | .039 | 1.000 | -.092 | .117 |
|  | 4 | .013 | .055 | 1.000 | -.132 | .157 |
| 4 Red | 1 | -.446^*^ | .086 | .000 | -.675 | -.218 |
|  | 2 | -7.013E-5 | .051 | 1.000 | -.135 | .134 |
|  | 3 | -.013 | .055 | 1.000 | -.157 | .132 |

Note. ^a^Based on estimated marginal means, ^b^Adjustment for multiple comparisons: Bonferroni. * The mean difference is significant at the .05 level.

Table 6. Pairwise comparisons of motorists’ estimate of other motorists’ tendency to drive/stop in the cycle lanes pictured

| (I) colour | (J) colour | Mean Difference (I-J)^a^ | Std. Error | Sig.^b^ | 95% Confidence Interval for Difference^b^ | |
| --- | --- | --- | --- | --- | --- | --- |
|  |  |  |  |  | Lower Bound | Upper Bound |
| 1 Uncoloured | 2 | .525^*^ | .077 | .000 | .321 | .730 |
|  | 3 | .656^*^ | .092 | .000 | .413 | .899 |
|  | 4 | .705^*^ | .101 | .000 | .437 | .974 |
| 2 Blue | 1 | -.525^*^ | .077 | .000 | -.730 | -.321 |
|  | 3 | .131^*^ | .045 | .025 | .010 | .251 |
|  | 4 | .180^*^ | .063 | .028 | .012 | .348 |
| 3 Green | 1 | -.656^*^ | .092 | .000 | -.899 | -.413 |
|  | 2 | -.131^*^ | .045 | .025 | -.251 | -.010 |
|  | 4 | .049 | .063 | 1.000 | -.118 | .217 |
| 4 Red | 1 | -.705^*^ | .101 | .000 | -.974 | -.437 |
|  | 2 | -.180^*^ | .063 | .028 | -.348 | -.012 |
|  | 3 | -.049 | .063 | 1.000 | -.217 | .118 |

Note. ^a^Based on estimated marginal means, ^b^Adjustment for multiple comparisons: Bonferroni. * The mean difference is significant at the .05 level.

Table 7. Pairwise comparisons of cyclists’ estimates of motorists’ tendency to drive/stop in the cycle lanes pictured

| (I) colour | (J) colour | Mean Difference (I-J)^a^ | Std. Error | Sig.^b^ | 95% Confidence Interval for Difference^b^ | |
| --- | --- | --- | --- | --- | --- | --- |
|  |  |  |  |  | Lower Bound | Upper Bound |
| 1 Uncoloured | 2 | 1.317^*^ | .138 | .000 | .948 | 1.686 |
|  | 3 | 1.480^*^ | .167 | .000 | 1.037 | 1.924 |
|  | 4 | 1.540^*^ | .199 | .000 | 1.011 | 2.069 |
| 2 Blue | 1 | -1.317^*^ | .138 | .000 | -1.686 | -.948 |
|  | 3 | .163 | .100 | .628 | -.104 | .431 |
|  | 4 | .223 | .142 | .698 | -.154 | .600 |
| 3 Green | 1 | -1.480^*^ | .167 | .000 | -1.924 | -1.037 |
|  | 2 | -.163 | .100 | .628 | -.431 | .104 |
|  | 4 | .060 | .129 | 1.000 | -.284 | .403 |
| 4 Red | 1 | -1.540^*^ | .199 | .000 | -2.069 | -1.011 |
|  | 2 | -.223 | .142 | .698 | -.600 | .154 |
|  | 3 | -.060 | .129 | 1.000 | -.403 | .284 |

Note. ^a^Based on estimated marginal means, ^b^Adjustment for multiple comparisons: Bonferroni. * The mean difference is significant at the .05 level.
